# The case for eliminativism about words

**DOI:** 10.1007/s11229-022-03846-0

**Published:** 2022-08-19

**Authors:** Nick Tasker

**Affiliations:** Hertford College, University of Oxford, Oxford, UK

**Keywords:** Words, SLEs, Eliminativism, Illusions, Ordinary objects, Mind-dependence, Slurs

## Abstract

Words are ubiquitous and familiar, and the concept of a word features both in common-sense ways of understanding the world, and in more theoretical discourse. Nonetheless, it has been repeatedly argued that there is no such thing as words. In this paper, I will set out a range of arguments for eliminativism about words, and indicate the most promising responses. I begin by considering an eliminativist argument based on the alleged mind-dependency of words, before turning to two challenges arising from linguistic theory in the Chomskian tradition. The first of these is issued by Rey in a number of places, including in his recent book (Rey, 2020). The second is Collins’s ( 2010, 2021a) argument based on the alleged explanatory redundancy of words. I will also consider an eliminativist challenge based on the difficulty of providing existence and persistence conditions for words. One general lesson which emerges is that these eliminativist arguments, if they work at all, could be turned against a whole swathe of non-linguistic objects; in other words, the case for eliminativism about words is no stronger than the case for eliminativism about ordinary objects in general.

## Introduction

Words are ubiquitous and familiar, and the concept of a word features both in common-sense ways of understanding the world, and in more theoretical discourse. Nonetheless, it has been repeatedly argued that there are no such things as words. In this paper, I will set out a range of arguments for eliminativism about words, and indicate the most promising responses. I begin by considering an eliminativist argument based on the alleged mind-dependency of words, before turning to two challenges arising from linguistic theory in the Chomskian tradition. The first of these is issued by Rey in a number of places, including in his recent book (Rey, 2020). The second is Collins’s (2010, 2021a) argument based on the alleged explanatory redundancy of words. I will also consider an eliminativist challenge based on the difficulty of providing existence and persistence conditions for words. One general lesson which emerges is that these eliminativist arguments, if they work at all, could be turned against a whole swathe of non-linguistic objects; in other words, the case for eliminativism about words is no stronger than the case for eliminativism about ordinary objects in general.

## The argument from mind-dependence

Philosophical theories of words are typically situated within what Guala (2007, p. 956) calls “the Standard Model of Social Ontology” (SM) which grounds aspects of social reality in human beliefs, attitudes and intentions. One finds variations on themes associated with the standard model entering into the literature on words in Barber (2006, 2013), Cappelen (1999), Kaplan (1990), Stainton (2014), and Szabo (1999) According to many such theories, words are socially/mentally constructed artefacts; they are (at least partly) mind-external entities which nonetheless depend constitutively for their existence on human representations.

My own preferred approach falls within this tradition, and draws inspiration from Thomasson’s (2003, 2007, 2014) theory of intentional artefacts. Briefly, a speaker intends their utterance to have certain linguistic properties. These may include semantic, syntactic, morphological, and phonological or orthographical properties, among others. The resulting utterance has to meet certain conditions in order for those linguistic intentions to be fulfilled. It meets these conditions if and only if it is such that it makes the speaker’s linguistic intentions recognisable to other speakers. When the speaker’s intentions are recognisable, the intended linguistic properties are successfully projected onto an utterance.^1^ This account faces a number of specific obstacles, notably including worries about whether the cognitive states responsible for linguistic behaviour can appropriately be described as involving *intentions* in the relevant sense. However, I sketch the account for illustrative purposes, and do not undertake here to defend it from such specific objections. Instead, my concern is to consider eliminativist arguments which threaten any account of words situated within SM.

One such argument focusses on the constitutive role which human attitudes and representations play in the metaphysics of social entities. Elder (2007) puts the point this way:

The intentions of the artisans among us, and the uses to which the rest of us put their products, simply seem to play too lightly over the surfaces of our material surroundings. It can seem unbelievable that matter upon which such intentions and uses are focused thereby comes to be a material object different in its essential nature from what would exist in its place, in the absence of such focusing. Thus it is very widely agreed that in the world which serious ontology inventories, there are no artifacts…Their careers are projected by people onto indifferent materials. (Elder, 2007, p. 33)

Underlying Elder’s elegant metaphor is the thought that mind-independence provides an essential criterion of realist commitment. As Devitt notes:

The general doctrine of realism about the external world is committed not only to the existence of this world but also to its ‘mind-independence’: it is not made up of ‘ideas’ or ‘sense-data’ and does not depend for its existence and nature of the cognitive activities and capacities of our minds. (Devitt, 2005, p. 768)

Such a criterion threatens realism about not only words, but perhaps all artefacts and social entities: within SM, phenomena such as chairs, money, artworks, recessions, and governments are all treated as mind-dependent in some sense. We should nonetheless like to treat at least some such phenomena as real in a way that Santa Claus and ghosts are not. Moreover, many such mentally/socially constructed entities are the subject of fruitful inquiry in the social sciences. So a minimal response would just point out that the present argument does not reveal words to be worse off, ontologically speaking, than a whole swathe of ordinary objects. But in fact it is worthwhile to respond to the objection directly, and on behalf of social entities in general.

Let us distinguish two kinds of mind-dependence. First, gold cubes or roentgenium atoms are *causally* mind-dependent, since nature does not in fact produce them without the intervention of human ingenuity. Within SM, wedding rings, chairs, and words are mind-dependent in a stronger sense, since they depend *constitutively* on the intentions or attitudes of their creators and users. For example, according to Thomasson’s theory of artefacts, an artefact is partially constituted by its creator’s intention.

Causal mind-dependence is no obstacle to existence, for roentgenium atoms are ontologically unimpeachable. So perhaps the problem is constitutive mind-dependence. But why should being partially or wholly constituted by mental states be any bar to ontological commitment? Khalidi (2015) points out that constitutive mind-independence should not be considered an essential criterion of existence, noting that beliefs, desires, and emotional states are mental entities and are therefore themselves constitutively mind-dependent. To this list, I would add the representational states posited in cognitive science, which could be accepted as real even by folk-psychological eliminativists. In short, human minds have a place in the natural world. That being so, the fact that an entity is partially constituted by human attitudes or intentions should be no obstacle to one’s taking a realist attitude to that entity, any more than a biological organism’s constitutive dependence on chemical entities is an obstacle to realism about biological organisms.

In response, it might be suggested that there is an important sense in which beliefs and other mental states are unlike chairs and words. To be sure, any mental state is constitutively dependent on a mind, but no one need have any attitude towards that mental state for it to exist. For example, if I-language states (see, e.g. Chomsky, 2000) are real, then they existed before anyone had any beliefs about them. But chairs and words are not like this (according to SM): a particular concrete object can only be a chair, or a token of a word, if someone has the right kind of attitude towards that very object, or at least towards that kind of object. This is problematic, the objection continues, because we are perilously close to saying that chairs and words exist purely because they are believed to exist. This gets the project of ontology backwards, and it also seems to rule out the possibility of falsely believing that something exists, the luminiferous ether, say, even though it doesn’t.

In reality, no one thinks it is sufficient for some object to exist that someone believes it does. To take a specific example, according to Thomasson’s theory of artefacts, the creation of a new artefact token requires that the artificer’s intention be successfully realised. In many cases, this requires the imposition of certain physical properties: nothing can be a knife unless it is sufficiently rigid, sharp, etc. In other cases, it is at least necessary that the artificer’s intention be *recognisable*. One cannot create money or words by intention, where those intentions are recognisable to no one. So in countenancing realism about socially constructed entities, we need not be guilty of the egregious error suggested in the last paragraph.

Khalidi (2016) argues that no distinction between mind-dependence and mind-independence provides a criterion for realist commitment. To illustrate, the non-existence of phlogiston and fairies has nothing to do with mind-dependence. It’s just that the world doesn’t contain anything corresponding to such concepts. Instead of seeking a criterion of existence in relation to mind-independence, Khalidi (2016, p. 242) recommends a “causal criterion of reality” according to which “something is real if (and only if) it is capable of making a causal difference.”^2^ If that is right, then the mind-independence of words is no obstacle to realism. Instead, we should ask whether words enter into causal explanations of matters of fact. That is one of the major themes of this article, but it must wait until section four.

## The argument from illusion

The most sustained attacks on realism about words have come from theorists within the Chomskian tradition in linguistics and philosophy. Rey (2005, 2006a, 2006b, 2008, 2020) is a prominent example, defending an eliminativist position regarding words, sentences, morphemes and other supposed linguistic items, which he calls *Standard Linguistic Entities* (SLEs). Ultimately, Rey’s position rests on considerations to do with explanatory redundancy, which are the subject of the next section. But he also offers a distinct argument, which I call *the argument from illusion*. This argument is our immediate concern.

Rey (2006a, p. 244) announces that “one of the linguist’s arguments for the nonexistence of SLEs is in a way extremely short.” Its strategy is to highlight some linguistic property which an utterance must have if it is to be an instance of a given SLE, and then to claim that no utterances (or inscriptions) have those features.

For example, Rey assumes that language users represent utterances of a given word as having a certain acoustic structure. In the case of the word ‘cat’, the idea would be that a speaker represents an utterance of that word as being composed of three distinct speech sounds in a linear temporal order. The problem, says Rey, is that our word-utterances just don’t have the acoustic properties we represent them as having. These claims receive support from a wealth of research in phonology and acoustic phonetics: phonologists talk of a level of phonological mental representation which organises perceptions of the speech signal into linear sequences of words, themselves made up of discrete speech sounds, while acoustic phoneticians affirm that this structure is not itself in the speech signal, which is chaotic and variable.^3^ The upshot is that the way we mentally carve the space of utterances into word kinds does not faithfully and naively track acoustic invariances.

To take another example, Rey (2005) considers the sentence ‘John seems to Bill to want to help himself’. Rey draws attention to two features of this sentence, as analysed in current linguistic theory: first, the sentence has a hierarchical structure (of the kind depicted in phrase structure diagrams), and, second, certain positions in that structure are occupied by phonologically null elements like PRO. But, Rey thinks, an actual utterance of the sentence doesn’t have these characteristics. Nothing in the acoustic profile of the utterance corresponds to that hierarchical structure, and no acoustic event can be said to manifest PRO:

“[N]ot only is there an elaborate tree structure…, there are also “empty” categories: trace (*t*_1_) and PRO…[D]oes anything I actually produced in space and time have the above structure? I think not” (Rey, 2005, p. 404).

SLEs are thus illusions, thinks Rey: it appears to us that there are such things as words and sentences, but really there are none. Luckily, communication succeeds because we all suffer from similar illusions in similar ways. Nor is the current practice of linguistic science impugned, since linguistics studies speakers’ mental representations of SLEs, not SLEs themselves. The non-existence of SLEs is no more an impediment to linguistics than the non-existence of ghosts is an impediment to the psychological study of belief in paranormal phenomena.

At a first pass, we might set out the general form of Rey’s argument as follows:

1. A competent speaker represents an SLE, X, as having property Y.
2. No mind-external entity produced by the speaker has property Y.
3. Therefore no mind-external entity produced by the speaker is an instance of X.

As stated, this schema is not set up to produce valid arguments. This is because (P1) is not strong enough. After all, we misrepresent real objects all the time. From the fact that the stick looks bent, we may not conclude that the stick does not exist! Let us therefore supply a suitably strengthened replacement for (P1):

1. Having property Y is essential to being an instance of SLE, X.
2. No mind-external entity produced by the speaker has property Y.
3. Therefore no mind-external entity produced by the speaker is an instance of X.

In the foregoing discussion, we have seen some example values for the X and Y variables. Each particular argument produced in this way yields an eliminativist conclusion about a particular sentence or word. How do we go from here to the general conclusion that there are no words? Rey doesn’t really tell us, but, plausibly, the idea is that as soon as one or two specific examples are provided, many similar arguments can be easily generated. That is, for any given word, one can easily find some linguistic property which the word is supposed to have, but which no utterance has.

One does not find instances of (P1*) being specifically asserted in Rey’s writings, at least not in so many words. It is nonetheless reasonable to characterise Rey’s argumentative strategy as involving instances of (P1*). First, he does characterise the argument as involving “the central properties associated with a term” (Rey, 2020, p. 303). Second, he explicitly appeals to instances of (P2) (as shown by the quotation above), and without a corresponding instance of (P1*), such an appeal will not yield a valid argument for Rey’s advertised eliminativism. Third, as I will show, Rey’s reliance on (P1) is not in doubt and, given his background assumptions, one can argue that (P1) entails (P1*), at least for some values of X and Y.

Rey’s (2005) article begins with a reminder of his commitment to the “causal/computational-representational theory of thought”, and his then recent dispute with Chomsky over the correct interpretation of generative linguistic theory. According to Rey, the task of the linguist is to reveal the *intentional content* of language users’ mental representations of language: “the most natural way to understand Chomskian linguistics is in fact as being about the intentional contents of those computations’ representation” (Rey, 2003, p. 141). This is a significant departure from Chomsky’s own understanding of the theory. The latter maintains that “The notions of “representation” and intentionality that [Rey] has in mind do not enter into such work, apart from passages that provide informal motivation” (Chomsky, 2003). It is thus part of Rey’s distinctive understanding of linguistic theory that when linguists analyse sentences in the way depicted in phrase structure diagrams, what they are doing is characterising the content of a competent speaker’s mental representation of the speech signal. Similar remarks apply to phonological analysis. As Rey puts it:

[I]nstructions issue from speakers’ phonological systems to produce certain SLEs, and these, instructions cause various motions in their articulatory systems which in turn produce various wave-forms in the air. These wave-forms turn out, however, not to reliably correspond to the SLEs specified in the instructions. (Rey, 2005, p. 405)

Here, we see clearly that the problem is supposed to be that the wave-forms do not satisfy the *speaker’s* mental instructions. The result, according to Rey (2005, p. 405), is a “perceptual illusion”.

How do we go from the claim that a speaker represents a signal as a sentence containing PRO, to the claim that containing PRO is an essential characteristic of that sentence? Here we must remember that Rey is approaching these matters from a biolinguistic perspective according to which the ultimate source of linguistic properties is the human mind/brain. As Rey (2008, p. 186) puts it, “[e]ven if acoustic blasts were to have linguistic properties, they would have them…in virtue of psychological facts.” In other words, it is speakers’ mental representations which determine the linguistic properties of SLEs. This might be taken to mean that a speaker is protected from error about the linguistic properties of the SLEs they represent. Consequently, if a speaker’s conception of an SLE depicts it as containing PRO, then anything which is a genuine instantiation of that SLE must contain PRO.

We could reject the head-first biolinguistic approach. Then we could say that instances of ‘cat’ exist despite the fact that none of them have the acoustic structure we represent them as having. This would be like finding out that cats are robots: we have been deceived about the true nature of some familiar kind of object, but we managed to refer to them despite our misconceptions. Rey will object that this rejection of the head-first approach ignores the direction of explanation in linguistics.

Alternatively, we could acquiesce to the head-first approach but resist the essentialising move. Even if it’s true that speaker representations are the source of linguistic properties, why conclude that certain linguistic properties are essential to a given SLE? Indeed, it seems likely that words and sentences do not have essences. This conclusion receives support below when the difficulty of providing a theory of word individuation is discussed. Miller (2021) has gone further, arguing that essentialism for words must be wrong because it is part of the nature of words that they may change over time. In place of an essentialist model of words, Miller has argued that words are homeostatic property clusters. If so, then no instance of (P1*) is true. The trouble with this suggestion as a response to Rey is that he can run a version of his argument for just about any linguistic property you care to think of. Even if words are homeostatic property clusters, you still need a given acoustic blast to have some package of linguistic properties, and Rey will deny that that package of properties is genuinely instantiated by the blast.

The real weak point in Rey’s argument is (P2). To be sure, utterances don’t have their semantic, syntactic, and phonological properties purely in virtue of their intrinsic, acoustic properties, but this doesn’t entail that they don’t have them at all. A Martian scientist couldn’t determine a coin’s monetary value by studying its physical composition, but that doesn’t mean its monetary value is an illusion; its having that value has something to do with its provenance, and the way it is regarded by members of a community. In sum, although we cannot locate linguistic properties among the intrinsic properties of utterances, we cannot conclude that our utterances are not instances of SLEs, since linguistic properties could be relational features of utterances. The various theories of words within SM are attempts to turn this suggestion into theory.

According to Rey, the problem with such “social, response-dependent” accounts of SLEs is their failure to recognise that the arrows of linguistic explanation point inwards towards the internal linguistic capacity of individuals: “the underlying error is a failure to appreciate the important shift of the explanatory locus in modern linguistics, from external objects to internal conceptions” (Rey, 2008, p. 177). But to make this reply is to shift towards a different argument, one akin to the argument from explanatory redundancy, discussed in the next section. It’s fine to rest one’s case on the explanatory redundancy argument, but the argument considered above is a different argument, and one Rey has made repeatedly.

He makes it again in his (2020) book, though some of the details are different. In this recent discussion, Rey indicates that the eliminativist argument turns on the following principle:

*Property Preservation*: the central properties associated with a term should be properties of a scientifically identifiable phenomenon (object/state/event). (Rey, 2020, p. 303)

Satisfying this principle is treated as a necessary condition for a term to be considered as genuinely referring to a real entity or kind. In Rey’s view, many of our terms for ordinary objects *do* satisfy this condition:

[T]alk of houses, tables, chairs, trees and rivers, cats and dogs, properties of fluidity and elasticity, all seems to refer to phenomena that can be pretty stably identified with physical phenomena across speakers and contexts. A specific house can be identified with a specific physical structure that is used for habitation as it was intended to be. (Rey, 2020, p. 303)

The idea is that the term ‘house’ is associated with a set of physical conditions which are actually satisfied in some instances. Specifically, a house is a physical structure of a certain sort with physical people living inside it. Rey is aware that there will be borderline cases of houses, but he doesn’t take that to undermine the existence of houses. His point is that there are at least some clear cases of objects which satisfy the physical conditions for being a house. A little later, he makes a similar point about cars:

Cars, like (purported) linguistic entities are artifacts, arguably tokens of types, produced by human beings with specific intentions. But I submit it is absolutely crucial to the *explanation* of why a car is so reliable that it in fact has (or realises) a certain causal structure: the pistons fit snugly into the cylinders, so that, when the gas is ignited by the sparks from the plugs, they are pushed down with sufficient force to turn the crankshaft, and so forth. Most importantly, the standard properties of a car are the properties of this physical object with this complex causal structure. (Rey, 2020, p. 308)

The phrase “the *explanation* of why a car is so reliable” is puzzling: what we’re out to explain is not the reliability of the car, but the fact the object is a car. Rey’s answer is that a car is a physical object with a certain intrinsic causal structure, and the object in question happens to have that structure.

We have two examples of artefacts which qualify as real, by Rey’s lights: some houses and some cars. Why doesn’t he think words should qualify as real? The principle Rey calls ‘*Property Preservation*’ requires that for a term to have real objects in its extension, it must be that some “scientifically identifiable phenomenon” satisfies the (crucial parts of the) descriptive condition associated with the term. But the alleged challenge is *not* that acoustic blasts fail to be “scientifically identifiable”: a particular car, or a particular house—considered as an amalgam of atoms—is no more or less “scientifically identifiable” than a particular acoustic blast; an acoustic blast is a perfectly respectable, detectable, physical phenomenon. Rey’s objection is that SLEs are associated with descriptive conditions which are in fact not met by acoustic blasts. That is, competent speakers represent acoustic blasts as possessing crucial linguistic properties such as being segmented into discrete phonological units; those representations impose physical conditions on the acoustic blasts, conditions which are just not met (Rey, 2020, pp. 308–311). This is the same argument as in Rey’s early papers on the topic, so the objections canvassed above still apply.

Rey’s discussion of houses and cars might give the impression that he is covertly deploying a stronger principle than *Property Preservation*, such as the following:

*Physical Realisation*: if a descriptive term has any objects in its extension then it must be the case that (i) the descriptive condition associated with the term specifies a certain intrinsic causal structure the possession of which is sufficient for falling within the extension of the term, and (ii) some physical object possesses that structure.

Such a principle would entail eliminativism about SLEs, but it has little to recommend it. It entails eliminativism about a swathe of ordinary objects. Consider two gold rings which are intrinsic duplicates of each other, but where one is a wedding ring, and one is not. What makes something a wedding ring is not any physical, causal structure intrinsic to the ring, but the way it is regarded. Or consider pawns in chess. Bloom (1996) points out that there is no particular physical profile associated with a pawn. At a pinch, even a penny can be a pawn:

Note that one does not have to do anything to the penny for it to become a pawn… [W]hat makes this penny a pawn (as opposed to a queen, say) is the mental state of the person who is considering the chess problem. (Bloom, 1996, p. 18)

If Rey justifies his SLE eliminativism by an appeal to *Physical Realisation*, then he must be an eliminativist about pawns, wedding rings, and other social entities whose characteristic properties are not among their intrinsic, physical properties. However, the argument Rey has repeatedly made relies only on the weaker *Property Preservation*. This principle does not entail eliminativism about social objects, and it does not undermine a theory of words as social objects. As mentioned, what remains of Rey’s SLE eliminativism is the complaint that words, externalistically construed, are explanatorily redundant. That is the topic to which we now turn.

## The argument from explanatory redundancy

Some philosophers and linguists in the Chomskian tradition allege that the explanations offered in linguistics do not require externalist notions of words and language. Collins (2010, 2021a) has provided the most persuasive formulation of the challenge. His discussion mostly targets language (externalistically construed) in general, but it is easy to see how his arguments apply to other linguistic entities such as words.

Collins’s account is embedded in the stance called *methodological naturalism* found in e.g. Chomsky (2000). This includes the view that since generative linguistics is a flourishing chapter of cognitive science, it should not be surprising if it ends up discarding pre-theoretic notions of language. This, after all, is how science often proceeds. To take an example, one may begin an inquiry into the nature of air, only to discover that air is a variable mixture of oxygen, carbon dioxide, nitrogen, and so on. In serious explanatory contexts, air drops out of the picture. Something similar is alleged to have happened as linguistic theory has explained aspects of language users’ linguistic performance (including acceptability judgements) in terms of their internal cognitive processes. What this enterprise yields is a rich internal landscape of mental structures which are on active duty in ordinary instances of language use. Among these structures, we find an internalist counterpart to the ordinary notion of a word:

According to [a Chomskian conception] words are mental objects. Think of a person as having something like a mental dictionary. The entries in this mental dictionary constitute a person’s “lexicon,” which includes various “lexical items”… [T]he lexical items of the linguist are (ideally) defined in terms of the innately specified features that make them up. That is, they are defined in terms of “phonological,” “formal,” and “semantic features.” The phonological features are those that, after mental computations, lead to the production of a sound, represented in the “phonetic” features at the “phonetic interface” (PHON). The formal features (N(oun), V(erb), A(djective), P(re(post)position)) and semantic features…lead, after mental computations, to the production of a specific meaning, represented in a configuration of features at the “semantic interface” (SEM). (McGilvray, 1999, pp. 95–96)

Such internal structures have an explanatory role in linguistic theory, and are deserving of ontological commitment, given the empirical success of such theories. But words and languages—externalistically construed—are simply not to be found among the posits or entailments of linguistic theory. As Collins puts it:

The crucial point is that [languages construed externalistically] do not offer properties that are either necessary or sufficient for the characterisation of the linguistic structures that enter into current explanations…The externalia are not necessary because linguistic structure can be realized in a wholly internal manner, as in private monologue. They are not sufficient because (i) the richness of linguistic structure far outstrips any external signature and (ii) the apparently unlimited heterogeneity of the externalia recruitable in linguistic performance does not admit generalizations mappable onto the linguistic categories…In other words, the relevant categories are invariant over external differences and so cannot be identified with externalia without eliding that which does remain invariant over the recruitment of externalia, viz., the cognition of the competent speaker/hearer. (Collins, 2010, p. 48)

Collins thinks we cannot identify linguistic externalia independently of our cognition: considered as acoustic entities, there is nothing that all and only the things judged to be instances of ‘cat’ have in common which determines that they have a certain phonological, syntactic, or semantic profile; the only thing that ties them together is the mental state which characterises language users who produce or consume them. At this point, one could claim that external objects somehow inherit the linguistic structures of speakers’ mental states, but Collins says such talk serves no explanatory purpose:

One can always claim the linguistic structure is external…but if the structure is identifiable only through the cognitive resources of the speaker/hearer…then the externalia lose any independence as a proper parameter in any serious explanatory practice. If the structure is to be depicted as genuinely external for our best science, then, lest it become an explanatory dangler, we should be able to identify it independently of the relation it bears to our cognition… [W]e must insist that the externalia be something more than a reflection of the cognitive design with which we have already credited the subject. (Collins, 2010, pp. 48–49)

Consider the frog which protracts its tongue in response to any small, dark, moving object. There is a mechanism internal to the frog which is triggered by a wide range of external objects, including flies, flicked pieces of gravel, or even devices which artificially stimulate the frog’s retina. One might begin by asking what it is about a fly or a piece of gravel, which triggers the response. But since no particular external condition is either necessary or sufficient to trigger the response, one’s attention is directed upon the sensorimotor systems internal to the frog. What is invariant in tongue-protractions is some internal mechanism, not any feature of the external environment, and explanatory progress is made by understanding the internal mechanism. We can go on to say, if we like, that in virtue of the way a variety of objects are capable of triggering that mechanism, those objects belong to the kind *looks-like-frog-food*, but this will add nothing of explanatory value. The various things which trigger the mechanism do not constitute a natural kind. Similarly, we can say, if we like, that linguistic significance is projected onto externalia, but the only possible reason for saying this is that language users conceive of those externalia as having the linguistic properties in question. No explanatory achievements are secured by saying that linguistic properties are projected onto externalia beyond those already secured by saying that language users are in a certain linguistic mental state.

It is tempting to see the point just elaborated as fitting into an overarching argument for eliminativism about words. That would be a misinterpretation of Collins’s aims, although certain of his remarks might encourage such an interpretation. It is nonetheless instructive to imagine what an eliminativist argument based on explanatory redundancy would look like. Stainton and Viger (2022, p. 262) attribute the following argument to Rey (2020):

1. Anything that really and seriously exists plays a role in a serious explanatory project.
2. Languages and their parts understood as external to the mind do not play a role in any serious explanatory project.
3. Languages and their parts understood as external to the mind do not really and seriously exist.

Stainton and Viger call (P1) the “Quinean Principle”. As those authors note, this principle is not very plausible. They first consider a strong reading of the phrase, ‘plays a role in a serious explanatory project’, which yields the following principle: “anything which really and seriously exists is a posit of “Galilean” sciences” (Stainton & Viger, 2022, p. 270). What is a Galilean science? Chomsky has often used the phrase ‘Galilean style’ to describe his philosophy of science (see Chomsky (1980/2005, p. 8) for his earliest published use of the phrase), and Rey (2020, pp. 16–19) discusses the approach approvingly. A Galilean science would be one which seeks to explain observational data in terms of the deeper natural structures and principles which underlie the data; on such a conception, it is sometimes justifiable to ignore certain data which run contrary to an otherwise explanatory theory. This is because the relation between the underlying structures and observed phenomena may be indirect, with observable anomalies frequently occurring due to complex interactions of multiple causes. As Chomsky puts it (in a published interview):

[P]hysicists “give a higher degree of reality” to the mathematical models of the universe that they construct than to “the ordinary world of sensation”…[I]t is the abstract systems that you are constructing that are really the truth; the array of phenomena is some distortion of the truth because of too many factors, all sorts of things. (Chomsky, 2002, pp. 98–99)

Taking these remarks literally, the word-eliminativist might be thought to reason as follows: the observable realm of inscriptions and wave-forms is a superficial distortion. What is real are the cognitive linguistic structures bestowed on humans as part of their biology.

Stainton and Viger object that the Quinean Principle—on its strong reading—just doesn’t fit the actual practice of most working scientists: it is so demanding, they say, that “the entities described in climatology, ecology, and neuroscience will not “really and seriously exist” and “even what contemporary physics *in fact* quantifies over might turn out to be unreal” (Stainton & Viger, 2022, p. 270).

To this objection I would add that the Galilean philosophy of science is emphatically not a metaphysical stance. Its wisdom is to recognise that certain observational data are correctly dismissed as having limited *epistemological* import with respect to a given theory. The fact that some observed event is not explained by a theory, or even seems at first glance to be at odds with the theory, may sometimes be discounted. This is because the event can be viewed as arising from the interaction of numerous phenomena, not restricted to the underlying mechanism targeted by the theory. Recognising this does not require us to regard the observed phenomenon as non-existent, as the strong reading of the Quinean Principle would have it; the underlying structures need not be regarded as more *real* than the flotsam and jetsam of the observable realm (though they may be more fundamental, and play a role in a wider array of explanatory contexts). When illustrating the Galilean philosophy of science, Chomsky (2002, p. 98) gives the following example about Galileo himself: “the data that he threw out were not minor. For example, he was defending the Copernican thesis, but he was unable to explain why bodies didn’t fly off the earth.” This inconvenient data point could safely be ignored, given the crucial pieces of data which *were* explained by the theory. But there is nothing wrong with the data point itself: it is a fact that bodies do not routinely fly off the earth. Similarly, one who considers linguistics to be a Galilean science may ignore certain performance errors in a subject’s verbal behaviour: that a subject utters some ungrammatical string need not mandate revisions to the theoretical description of that speaker’s linguistic competence; but none of this has any bearing on the ontological status of their utterance.

Stainton and Viger go on to consider a weaker version of the Quinean Principle, namely that “*anything which really and seriously exists plays a part in substantial accounts of why things happen*” (Stainton & Viger, 2022, p. 270). Even this weakened principle seems false, they note, since it still rules out objects which—while explanatorily not very useful—are perfectly inoffensive, such as sweet, yellowish Chardonnay, or the right hemisphere of a bowling ball. For Stainton and Viger, this is a mere aside, since they go on to argue, as I will also argue, that linguistic entities *do* play a part in substantial accounts of why things happen. In other words, even if we were to grant (P1) on its weaker interpretation, the argument above does not go through because (P2)—suitably adjusted to preserve validity—is false.

It would, however, be wrong to attribute the argument set out above to Collins. Although some of his remarks suggest that Collins is an eliminativist about words, his official position is more nuanced. The following quotation is particularly instructive:

[T]he internalist is free to say that there is English, French, etc.; her point is not that E-languages do not exist, but that they do not feature, as presuppositions or entailments, in the explanations of linguistic theory…E-languages are claimed to be otiose from an explanatory perspective, but one might continue to believe in them free of any commitment to their being on explanatory duty in any sense, much as one might speak of musical pieces without thinking that any explanation of fact requires commitment to musical pieces as such. (Collins, 2021a, p. 161)

This passage makes clear that Collins does not adhere to the Quinean Principle, even in its weaker form. When Collins says chairs, languages, and words are not naturalistically real, he is *not* committed to their unreality. For all he has set out to establish, we might go on to elaborate a theory of words as external artefacts. Such a theory might not even be false; it would just, Collins thinks, be pointless. That is, one might be able to find some entity corresponding to ‘cat’ or the French language—perhaps some monstrous composite of sound waves, ink marks, intentions, conventions, or abstracta. Such entities may exist (just as the scattered object consisting of the dome of St Paul’s Cathedral and the Mona Lisa exists) and may even be describable with enough ingenuity and patience. But doing so will contribute nothing to the explanatory power of any serious scientific theory. It would be an exercise which Chomsky (2000, p. 129) describes as “wheel-spinning”.

Collins (2010, 2021a) develops this position by addressing a variety of linguistic phenomena—such as grammatical acceptability, the unboundedness of language, cross-linguistic comparisons, or communication. In each case he argues that any apparent reference to linguistic externalia in the theoretical accounts of such phenomena is loose talk which can be rephrased in purely internalistic terms without loss of explanatory power. It is not my intention to contest Collins’s account of these phenomena. I do not deny, as Devitt (2006a) does, that the subject matter of generative linguistics is human cognition, or that its consequences for philosophy of language are yet to be fully appreciated. Instead, I would make a plea for linguistic pluralism, in which different explanatory tasks call for different kinds of linguistic entities, including mind-external ones.

Let me sketch the pluralistic landscape I have in mind. First, I grant the linguists their claims about the internal realm of linguistic cognition. There really are I-languages, and I-expressions, and these objects are in some sense the fundamental linguistic phenomena. But then, I take seriously the “projection of structure onto sounds/marks” (Collins, 2010, p. 50), a process in which mind-external objects such as acoustic blasts and ink marks are “invested with linguistic significance” (Collins, 2010, p. 48). Collins is happy to employ such locutions, while reminding us that they are explanatorily redundant. I treat such talk as expressing a useful insight. Personally, I would develop that insight along the lines indicated above, namely, as a theory of words as intentional artefacts which would be deserving of ontological commitment alongside I-languages and I-expressions. But my goal here is just to defend realism about words in general, not any particular social ontological theory of words.

One advantage of the pluralistic approach is that it would save common sense. We do ordinarily talk as if there are mind-external words, and a pluralistic account like the one just sketched means that such thought and talk is typically true. Thus, we are not forced to embrace an error theory about ordinary talk, according to which our ordinary claims about words and language are fundamentally misguided. That would be a radical and disruptive conclusion, given the familiarity and ubiquity of word-talk.

Collins affirms that his arguments do *not* impugn common ways of talking about language: “commonsense can continue on its merry way” (Collins, 2010, p. 49). What precisely does he mean by this? Does he mean that common sense talk of language is literally true (even if of no theoretical import), or does he just mean that it is harmless? His numerous comments, documented above, to the effect that words and languages might be “real in some or other sense (in the same way, perhaps, games or pieces of music might be)” (Collins, 2010, p. 48) suggest that the first interpretation remains a live option. If that is the path to be pursued, then there really are words and languages, externalistically construed, and a theory like the one sketched above is needed to make sense of that, as a piece of social ontology even if not as a piece of linguistics.

On the other hand, when Collins (2010, p. 55) affirms that “[t]here are…not two sets of phenomena in the world, one for the philosopher, one for the linguist”, one suspects that he does not regard ordinary language talk as being made true by the real existence of words and languages. What are the options, short of embracing the error theory? One could argue that although ordinary word talk is true, it is not made true by the existence of words: instead, the truthmakers for word-talk could include many and various cultural factors.^4^ Collins (2021b) has defended a view along these lines to account for truths in fiction. This is distinct from a more traditional strategy, which would be to claim that ordinary word talk can be paraphrased into truths. This can be defended only if relevant and true paraphrases are forthcoming. Ideally, we would have a general recipe for providing such paraphrases, just as we could provide a general recipe for paraphrasing bachelor-talk: *replace ‘bachelor’ with ‘unmarried man’*. I’m sceptical that such a recipe could be provided, not least because many particular pieces of ordinary word-talk seem to resist paraphrase into internalistically acceptable vocabulary. Consider the following examples:

(i) *Many modern English swear words come from Anglo-Saxon English and are less offensive today than they were fifty years ago.*
(ii) *The King James Bible uses words which were archaic even at the time of publication.*
(iii) *The word ‘controversy’ should be pronounced with the stress on the first or second syllable, but not the third or the fourth.*

Such talk raises numerous problems for the provision of internalistic paraphrases. Sentence (i) purports to refer to sets of English or Old-English words. It is hard to see how to construct a set of internal lexical items which corresponds to the words of English, without reference to external entities: even if one had a fix on the speakers of English, one cannot simply take the sum of their lexical items, because different speakers will have qualitatively different internal lexical items corresponding to one and the same English word, and because multilingual speakers will have internal lexical items from other languages. It is even harder to see how to isolate the set of English swear-words, for reasons set out below (in connection with slurs). A problem raised by sentence (ii) is how to make sense of a word being archaic, since it makes no sense to say that the translators of the King James Bible employed archaic internal lexical items. Sentence (iii) raises the issue of normativity. That a word *should* be pronounced in some way or other cannot be expressed in terms of language users’ internal states: even if a majority of the linguistic community (whose borders are settled I know not how) represent a word one way, that doesn’t mean that it should be pronounced that way. Moreover, the targets of linguistic prescriptivism include facets of linguistic performance which far outstrip the internal states of language users. Perhaps some of these difficulties can be resolved with sufficient ingenuity, but without a general recipe for paraphrasing word-talk, the claim that appropriate paraphrases exist cannot be convincingly established.

The foregoing discussion bears on whether word-eliminativism entails that there is something fundamentally wrong with ordinary ways of talking and thinking about language. But if we wish to make a strong response to Collins, or to strengthen our response to the strictly eliminativist version of the argument from explanatory redundancy, we need to show that linguistic externalia do enter into causal explanations of matters of fact. That is the task to which we now turn.

As examples of scientific disciplines concerned with linguistic externalia, Stainton (2014) mentions clinical linguistics, computational linguistics, dialectology, discourse analysis, educational linguistics, forensic linguistics, historical linguistics, lexicography, and pragmatics. Stainton and Viger give a pair of specific examples:

Why are certain Francophone populations in Northern Ontario subject to high unemployment rates? Sociophoneticians might explain this, in part, in terms of microvariation in pronunciation in non-prestige dialects of French. Why do some multilingual patients recover English better than others do after an aphasia-inducing stroke? Speech language pathologists might explain this in terms of which other languages the person knows and the order of their acquisition…[A]sserting that all of this can be readily explicated via sets of I-languages is a massive promissory note. (Stainton & Viger, 2022, pp. 270–271)

The internalist is likely to contest these examples. Taking the sociolinguistic case, the claim will be that the only kinds of entities on explanatory duty are psychological ones. The fact that someone is refused a job is adequately explained (we are supposing) by the way their speech is *perceived* by the interviewer. The candidate is believed to have produced a linguistic entity of a certain type and it is believed that production of such entities makes someone a bad candidate. From a causal-explanatory point of view, it does not matter whether the candidate really has produced an entity of that type, or whether doing so really makes someone a bad candidate.

A possible rejoinder is that the present response does too much, and wrongly suggests that many ordinary objects are causally impotent. This is because a whole class of artefacts achieve their characteristic functions in virtue of the way they are regarded. Consider, for example, a foot-high fence around a private garden. By the reasoning just adduced, we can argue that the fence is not causally responsible for preventing trespass. After all, it doesn’t matter whether or not the wooden structure is a fence or not; all that matters is that would-be intruders regard it as one. Perhaps that *is* the right thing to say about foot-high fences. But in that case we must recognise that what is under discussion in this section is not a targeted attack on linguistic externalia, but a much broader attack on social entities. What we say about words and foot-high fences we must also say for traffic lights, price tags, police tape, wedding rings, trophies and medals, uniforms, crucifixes, churches, monarchs’ crowns, money, gang tattoos, war memorials, and so on.

Let’s now take a different example, adapted from a recent journalistic source: *the council worker was fired because she used the N-word*. The internalist will insist that the apparent reference to an external word—in this case a slur—is just a *façon de parler*. What really got the worker fired was the fact that she employed her linguistic capacity to externally express the lexical item [N*****].^5^ The trouble with this suggestion is that, while it may work as a description of the individual case, the strategy does not allow us to make certain true generalisations.

To see this, note first that we want to be able to say that the cause of the council worker’s sacking has something in common with a litany of other speech acts involving the same slur. On the face of it, what such events have in common is that they all feature uses of the N-word. It might be objected that there is no need to appeal to the real existence of the N-word; instead, ‘the N-word’ is merely a useful label for a variety of heterogeneous phenomena. But this is to ignore the fact that there is something which is invariant across all the various speech acts in which that slur is apparently employed, namely, an entity with some reasonably uniform cluster of causal powers, chiefly the power to cause offence, to intimidate, to denigrate people of colour, to recall a painful history of kidnap and enslavement, to normalise racist attitudes, and to situate the speaker within a culture of white supremacy.

This is not to deny that the slur has different effects in different contexts: consider the different reactions to its use by a white person as a deliberate insult, to reappropriated uses of the word by people of colour, or to mentions of the word in a sociolinguistics classroom. But we should not be surprised to find that the causal regularities which attend our linguistic behaviour are not strict regularities. To put the point in terms which are familiar to participants in the present debate, the precise effects of a use of the N-word are interaction effects, depending not only on the core causal powers of the slur, but also on the social status of the speaker, their intentions, their audience, and many other factors. Nonetheless, the core causal powers of the slur (to offend, to denigrate, etc.) are always on active duty. Taking the example of reappropriated uses, it is precisely because of the slur’s shocking power and history that it can become a powerful symbol of camaraderie. Similarly, mentions of the word in a sociolinguistics classroom may elicit more intellectual curiosity than moral outrage, but this curiosity is in large part due to the word’s powers to denigrate and offend; and let us not forget that even such mentions are liable to cause intimidation and offence.

So the cause of the council worker’s sacking has something in common with countless other racist speech acts, namely the N-word, a social entity with a distinctive cluster of causal powers. The internalist cannot couch such generalisations in purely internalistic terms, as the suggestion above has it. This is because the causal powers associated with the N-word have little to do with the corresponding internal lexical items in individuals’ mind/brains. As we move from speaker to speaker across continents and centuries, the relevant internal lexical items will exhibit huge variability in their phonological, syntactic, and semantic properties. Similarly, an alien or AI which processes language in a radically different way could use the slur with the same effects as when humans use it. Speakers’ beliefs and intentions will also exhibit variability: we can imagine a naïve and non-racist speaker who discovers the word for the first time in an old novel but has no idea of its meaning; if such a speaker goes on to use the word, it is likely to be harmful and offensive in all the usual ways. Conversely, we can imagine a Twin-Earth community which happens to have I-languages which are intrinsic duplicates of ours, but no racism or racial inequality. It’s conceivable that in such a community, externalisations of the lexical item [N*****] would not be slurs, and would not have the causal profile associated with the word in our community.

The present argument is that slur words are entities with a causal profile which is independent of any speaker’s internal linguistic state. Similar remarks apply to swear words, shibboleths, slang, liturgical terms, legal phrases, official titles, etc. What such examples illustrate is that explanations of certain social phenomena do require reference to words as mind-external entities. Some of these explanations are of an everyday variety, while others fall within the domain of sociolinguistics or other social sciences. Let us briefly take one more example.

Readers of the King James Bible are typically impressed by the gravity of its style. This widespread psychological effect can be explained, in part, by the fact that it employs archaic words, including words like ‘verily’ which were archaic even at the time of publication. The internalist may reply that such explanations do not require the existence of genuinely archaic words: it is enough that readers *believe* there to be archaic words in the text. But this is not right. First, readers may not have such beliefs: one doesn’t need to believe that ‘verily’ is archaic for it to have its peculiar tonal effects. Second, if these effects *were* due to some kind of illusion, it’s hard to see why the illusion should be so widespread. Consider the kinds of ‘illusions’ that Rey attends to, such as perceiving word boundaries or perceiving an utterance as containing PRO. It is no surprise that such effects are widespread since they arise from features of our perceptual systems and language faculty which are properties of humans as a species. What is the equivalent explanation for the widespread ‘illusion’ that the King James Bible contains archaic vocabulary? The obvious thing to say is that it is not an illusion. To the extent that people represent the King James Bible as containing archaic vocabulary, these representations are merely tracking the truth.

I’ve set out in this article to defend the existence of words against eliminativist challenges, and to show that words are at least no worse off, ontologically speaking, than many other ordinary objects. With respect to Collins’s challenge, enough has been said to achieve that modest aim. However, while I have also argued that words, externally construed, do play a role in explaining why things happen, there remains a significant challenge here, with wider ramifications for philosophy of language. Philosophers often make substantive claims about meaning, reference, language acquisition, knowledge of meaning, etc., and such claims are typically framed with reference to linguistic units such as words and sentences. What Collins has shown is that many such claims should be reformulated in internalistic terms. That doesn’t mean the game is up for externalist approaches to linguistic entities, but it does mean that externalism isn’t the only game in town.

## The argument from individuation

Under what conditions are two utterances utterances of the same word? One naïve response, that this is so if and only if they have the same acoustic form, is quickly defeated.^6^ Fluent speech is rapid and full of short-cuts: Wetzel (2008) notes that ‘extraordinary’ can be pronounced with six, five, four, three or even two syllables (“strornry”). A word can be spoken at high or low pitch, lisped, enunciated theatrically, sung, whispered, etc., and the acoustic properties of an utterance depend on the environment too (e.g. humidity). Pronunciation also varies with the age, sex, regional origin, physical condition, and native or non-native status of the speaker. Such factors ensure that two utterances of a word may vary wildly from an acoustic point of view. Consider also instances of formal coincidence between utterances of different words. It is frequently observed that English contains two ‘bank’ words (referring respectively to part of a river and a financial institution). The form-theoretic view provides no grounds for any such distinction. In addition, an utterance of ‘at all’ may contain a sound which duplicates that of an utterance of ‘tall’. There could also be cross-linguistic coincidences, where unrelated words in different languages sound the same. Finally, similar problems apply at the level of the sentence: saying the words ‘visiting relatives can be tiresome’ produces a sound which is ambiguous between two different sentences.

What other candidates are there for the essences of words? Meanings will not do: centuries ago the word ‘meat’ just meant *food*. Nor are syntactic properties of words essential: the phenomenon of turning nouns into verbs is ubiquitous in English (e.g. ‘to table a proposition’), and the result is intuitively not a new word but a new usage of it; the word ‘that’ can be used as a pronoun, a determiner, an adverb or a conjunction, but a given occurrence of ‘that’ will have only one of these syntactic properties.

Other proposals include Kaplan’s (1990) suggestion that two utterances are utterances of the same word just when they are connected by a continuous path of intentional transmission. Against Kaplan, three objections. First, on Kaplan’s view, our word producing intentions are self-fulfilling: it doesn’t matter if something goes wrong physiologically. Kaplan’s account predicts that an unintelligible, drunken slur can be a successful utterance of ‘otorhinolaryngologist’, even if the utterance has one syllable and no consonants. Hawthorne and Lepore (2011) made this objection, and Kaplan replied as follows:

Suppose someone has a terrible accident…[H]e intends to be uttering his name and telephone number. But all that comes out is a monosyllabic grunt...Does my view that the intention makes it so imply that the grunt is, in fact, an utterance of the name and telephone number? It need not… [T]he right thing to say in this case is that the injured person cannot speak…[H]e didn’t say what he intended to say, namely, his name and telephone number, he didn’t say anything at all. He cannot speak. (Kaplan, 2011, p. 519)

This won’t do. What the person cannot do is *say words*. The effect of their injury is that they are no longer able to do whatever one is required to do to say a word, over and above having the right intentions. That just proves that there *is* something involved in successfully saying a word other than having the right intentions.

A second objection comes from cases of fission: the words ‘skirt’ and ‘shirt’ have the same etymological root, but they remain different words. This conflicts with Kaplan’s claim that being part of a certain causal-historical chain is sufficient for being an instance of a given word. A third objection comes from cases of fusion: it is possible for a word to be invented more than once, just like calculus or the hand-axe. Languages as disparate as Catalan, Hungarian, and Tagalog have very similar ways of expressing gustatory pleasure (roughly ‘nam nam’). Suppose, as seems likely, that these expressions have no common ancestor. I would say a single word had been invented multiple times. This conflicts with Kaplan’s claim that being part of a certain causal-historical chain is necessary for being an instance of a given word.

The apparently intractable difficulty of providing a theory of word individuation raises concerns about the viability of a realist attitude towards words. After all, we are unable to provide solid answers to a host of reasonable questions: Is ‘bank’ (referring to a financial institution) the same word as ‘bank’ (referring to a feature of river geography)? Is ‘cyning’ (in Old English) the same word as ‘king’? Does a photograph of a text contain instantiations of words or just pictures of them? If I spell my name with fridge magnet letters, then jumble them up before putting them back in order, is the new token of my name identical to the old one? Perhaps these questions have determinate answers, but if not, then words and their instances lack determinate conditions of existence and persistence. This is a serious challenge to word realism.

The best thing we can say in response is that this is a familiar predicament: it is no easier to provide individuation conditions for chairs, wedding rings, and chess pieces; and individuation problems also afflict biological entities such as organisms and species. So if the problem of individuation leads to word eliminativism then it also leads to eliminativism about a range of social and biological entities too. As noted, Miller (2021) argues that words are homeostatic property clusters, a view which aims to secure realism about words while explaining their resistance to classical definition.

However, Hawthorne and Lepore suggest that words face paradoxes of persistence which set them apart from other ordinary objects:

Suppose x belongs to a community that uses a particular word, ‘happy’. Two communities c_1_ and c_2_ pass by x’s community and, by x’s lights, appear to pick up that word and return to their homelands with it. x has a description of this case that by her lights is extremely natural: c_1_ picks up that word and comes to pronounce it in one way, while c_2_ picks up the word but comes to pronounce it in a very different way—let us say ‘harpy’ and ‘hapry’. Suppose c_1_ and c_2_ come to attach different meanings to the relevant words. Again, this will have a natural description by the lights of x: c_1_ and c_2_ use the same word with slightly different meanings…Suppose the c_1_ users pick up the c_2_ uses of ‘hapry’ and the c_2_ users pick up the c_1_ uses of ‘harpy’. Members of both c_1_ and c_2_ will find it natural to think they are using two different words. Even if they learn that there is a common origin, this likely will not affect that judgment…So who is right?…[S]iding with one perspective seems bizarrely chauvinistic. [Eliminativism] offers a way out of the dilemma.” (Hawthorne & Lepore, 2011, pp. 483–484)

Despite Hawthorne’s and Lepore’s suggestion to the contrary, it is not obvious that this problem does set words apart from other social entities. Other repeatable artefacts—such as orchestral compositions, folk dances, culinary dishes, and so on—give rise to analogous problems: it is easy to imagine that the signature dish or dance of one community is modified in different ways in two corners of that community’s diaspora, and that the new variants are then shared across the diaspora, producing identity judgements which mirror those in the ‘happy’/‘harpy’/‘hapry’ case. However, someone might object that my examples of repeatable artefacts form a rather paltry list, and that one could be an eliminativist about folk dances and Rogan Josh without rejecting social entities in general. Moreover, it is not so easy to construct a Hawthorne and Lepore style fission case with a more ontologically respectable artefact-kind such as the combustion engine or mechanical clock.^7^ So while Hawthorne and Lepore style cases might encourage an eliminativism which is not confined to linguistic entities, they do not obviously lead to eliminativism about social entities in general. Consequently, it would be better to respond to the challenge directly.

From the perspective of the word-realist, what is threatening about Hawthorne’s and Lepore’s case is that it purports to show that different people can make conflicting but equally reasonable judgements about word-identity. Hawthorne and Lepore seem confident that x will judge that ‘happy’ = ‘harpy’ = ‘hapry’, and that members of c_1_ and c_2_ will judge that ‘harpy’ ≠ ‘hapry’, adding that to side with one perspective would be chauvinistic. However, the intuitions advertised in the thought experiment are not so clear-cut, stemming from the fact that the phonological and semantic changes which are supposed to have occurred are underspecified. How big are the changes in pronunciation? Do members of c_1_ and c_2_ pronounce ‘r’ the same way? Are the new meanings subtle variations within the same lexical field or radical departures? If the changes are minor then the proper response may be that ‘happy’ = ‘harpy’ = ‘hapry’, and that should be your view whether you are x or a member of c_1_ or c_2_. Hawthorne and Lepore think any member of c_1_ or c_2_ is bound to deny this since she uses both ‘harpy’ and ‘hapry’ *as if* they were different words. But in support of my position, one can imagine initially believing that ‘conTROversy’ and ‘CONtroversy’ are distinct words before coming to learn that they are one and the same. On the other hand, if the changes are much more significant then it may well be that ‘happy’ ≠ ‘harpy’, ‘happy’ ≠ ‘hapry’, and ‘harpy’ ≠ ‘hapry’, but, again, that should be your view whether you are x or a member of c_1_ or c_2_. A word can change over time, but there are limits to this tolerance: it may be true that our word ‘gay’, meaning *homosexual*, comes from the Frankish word ‘gahi’ meaning *pretty*, but even if it is, ‘gay’ and ‘gahi’ are not the same word.

## Conclusion

Philosophy is replete with arguments against the existence of ordinary objects, including causal redundancy arguments, sorites arguments, problems of material constitution, and others. It’s no surprise that similar arguments can be turned on words. The minimal conclusion I have aimed to justify is that word-eliminativism is no more plausible than a widespread eliminativism regarding ordinary objects. My response to the general difficulty of providing existence and persistence conditions for words rests on such a claim, though I have provided a direct response to Hawthorne’s and Lepore’s more specific fission argument. The mind-dependence which is characteristic of words—according to theories within SM—is also a feature of other ordinary objects, but in this case I have argued that the mind-dependence of an entity does not disbar it from ontological commitment. The other arguments considered here purport to justify eliminativism about words in particular. But Rey’s argument from illusion, if it establishes anything, establishes a non-specific eliminativism about social entities. In fact, it does no such thing, given that many social entities have their characteristic properties in virtue of their relations to people and societies, and not purely in virtue of their intrinsic physical structure. Rey’s case for SLE-eliminativism consequently collapses into the kind of explanatory redundancy argument discussed in section four. There I have tried to show that words are needed in a variety of causal explanations, as entities with distinctive causal powers which are independent of speakers’ internal linguistic states. Nonetheless, I do not contest claims made by Rey and Collins that many areas of linguistic inquiry are best pursued by ignoring and abstracting away from concerns with linguistic externalia. The consequence is a kind of word-pluralism, which forces us to admit that there is no single conception of words capable of accounting for common sense, along with the various requirements of philosophy, social science, and linguistics.

## Footnotes

1 Barber (2013, p. 974) makes a related proposal.

2 Given the difficulties posed by e.g. abstract objects, Khalidi notes that this criterion may not apply in all domains.

3 A small sample of relevant results from Fodor et al (1974)—also cited by Rey—will illustrate this claim. First, different acoustic signals can be heard as instances of a single speech sound. For example, the precise nature of the vowels in utterances of ‘da’, ‘ga’, ‘ka’, and ‘ba’ varies due to the articulatory effects of the preceding consonant. If a machine is used to synthesise vowel sounds associated with the presence of a particular preceding consonant, subjects hear the sound as containing the relevant consonant (Fodor et al, 1974, p. 292). Second, a single acoustic signal can be heard as an instance of distinct speech sounds. When a recording of ‘pi’ is edited to preserve only the initial consonant, and then spliced onto a recording of a different vowel such as ‘a’, the composite recording is consistently heard as ‘ka’ rather than ‘pa’ (Fodor et al, 1974, p. 295). Third, there is no segment-by-segment correspondence between a speaker’s phonological representation of a speech signal and the acoustic reality. For example, in American English, the middle consonants in ‘rider’ and ‘writer’ are indistinguishable from an articulatory or acoustic perspective. The acoustic cue which enables language users to distinguish utterances of these words is the length of the preceding vowel (Fodor et al, 1974, p. 292).

4 I am grateful to an anonymous referee from this journal for drawing my attention to this option.

5 Thanks to an anonymous referee for this point.

6 I focus on speech, but analogous considerations apply to writing: consider the effects of different typefaces, handwriting styles, careless handwriting, different spelling conventions, etc.

7 See Lowe (2014) for a defence of the view that technological machines deserve ontological commitment even if realism about ordinary artefacts succumbs to worries about their existence and persistence conditions.
